# The Use of Ecological Niche Modeling to Infer Potential Risk Areas of Snakebite in the Mexican State of Veracruz

**DOI:** 10.1371/journal.pone.0100957

**Published:** 2014-06-25

**Authors:** Carlos Yañez-Arenas, A. Townsend Peterson, Pierre Mokondoko, Octavio Rojas-Soto, Enrique Martínez-Meyer

**Affiliations:** 1 Biodiversity Institute, University of Kansas, Lawrence, Kansas, United States of America; 2 División de Posgrado, Instituto de Ecología A.C., Xalapa, Veracruz, México; 3 Red de Biología Evolutiva, Instituto de Ecología A.C., Xalapa, Veracruz, México; 4 Instituto de Biología. Universidad Nacional Autónoma de México, México City, México; Universidad de Costa Rica, Costa Rica

## Abstract

**Background:**

Many authors have claimed that snakebite risk is associated with human population density, human activities, and snake behavior. Here we analyzed whether environmental suitability of vipers can be used as an indicator of snakebite risk. We tested several hypotheses to explain snakebite incidence, through the construction of models incorporating both environmental suitability and socioeconomic variables in Veracruz, Mexico.

**Methodology/Principal Findings:**

Ecological niche modeling (ENM) was used to estimate potential geographic and ecological distributions of nine viper species' in Veracruz. We calculated the distance to the species' niche centroid (DNC); this distance may be associated with a prediction of abundance. We found significant inverse relationships between snakebites and DNCs of common vipers (*Crotalus simus* and *Bothrops asper*), explaining respectively 15% and almost 35% of variation in snakebite incidence. Additionally, DNCs for these two vipers, in combination with marginalization of human populations, accounted for 76% of variation in incidence.

**Conclusions/Significance:**

Our results suggest that niche modeling and niche-centroid distance approaches can be used to mapping distributions of environmental suitability for venomous snakes; combining this ecological information with socioeconomic factors may help with inferring potential risk areas for snakebites, since hospital data are often biased (especially when incidences are low).

## Introduction

Only a small percentage (10–15%) of ca. 3000 known species' of snakes is venomous, and thus potentially dangerous to humans [1]. However, in the tropics, snakebites are a significant cause of human mortality and morbidity, with important impacts on human health, as well as to economy through treatment-related expenses and loss of productivity [2]. Recent estimates suggest that at least 421,000 bites and 20,000 deaths occur worldwide from snakebite annually, possibly ranging as high as 1,841,000 bites and 94,000 deaths [3]. The most affected regions in the world are sub-Saharan Africa, Southeast Asia, and Latin America [3]–[5].

Despite the scale of effects on human populations, snakebites has not received much attention from national and international health authorities, and has now been categorized as a “neglected tropical disease” [6]. Diverse authors have studied the problem [3], [7]–[10]; however, most of these studies involve hospital records, and the representativeness of this information has been questioned [7]–[10]. According to Chippaux [8], prospective enquiries in randomly selected localities is preferable, but this procedure would be long and expensive. Hansson et al. [9] developed an index of potential underreported cases of snakebites using environmental, socioeconomics and health-care related variables, a valuable contribution towards a better understanding of snakebite incidence, but other aspects of the phenomenon may enrich the view, such as the specific identity, geographic distribution and abundance of venomous snakes involved in the incidents.

Ecological niche modeling (ENM) has been used widely in recent years to map potential geographic distribution of species', with reliable results [11]. Nevertheless, capacity of these models to inform about the distribution patterns of abundance is, in the best case, limited [12], [13]. Recently, however, a new method based on the distance to niche centroid (DNC) was proposed, that may offer a better understanding of how abundance is structured within the margins of species' distributions [14]. This method seeks a relationship between distance to the niche centroid and abundance; therefore, it can used as a measure of environmental suitability (ES), wherein conditions close to the niche centroid represent higher suitability, and therefore potentially higher abundance [14], [15].

The principal goal of this paper was to evaluate the utility of ENM and DNC approaches in combination with socioeconomic variables to infer potential risk areas of snakebites in the state of Veracruz, Mexico. We first modeled the geographic and ecological distribution of each venomous snake species' occurring in the region; then DNCs were calculated for each species based on the ENMs. We analyzed relationships between reported incidences of snakebite and DNC values. Finally, we built several models relating DNC of snakes and socioeconomic variables to snakebite incidence. We selected the state of Veracruz based on the next criteria: (1) it has the second highest snakebite rate in Mexico, with approximately 15% of the country's total fatal accidents per year [16]; (2) arguably the most dangerous snake in Latin America (*Bothrops asper*) is widely distributed in the state [17], [18]; and (3) three authors of this paper have been working and living in this region for many years, and hence know well the area and the snakes occurring there.

## Methods

### Study area

**Figure 1:**
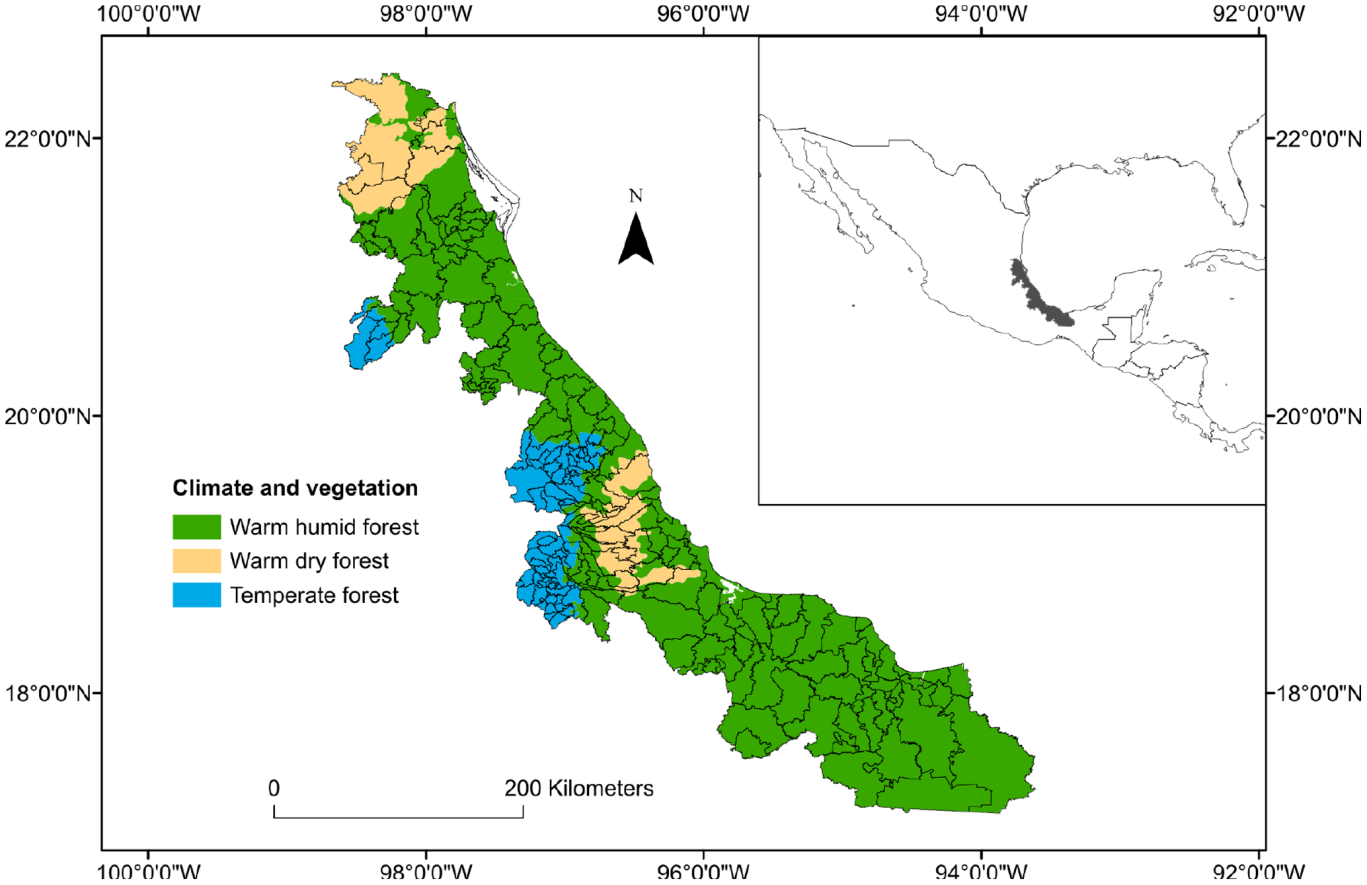
Climate, vegetation and topography of study area (Veracruz, Mexico). Black lines represent municipalities.

The state of Veracruz is located on Mexico's Gulf coastal fringe extending 745 km north to south, covering 72,420 km^2^ (3.7% of the total area of the country) (Figure 1). About 80% of the area has been transformed by expansion of the agricultural frontier and human settlements [19]. The state includes a long coastal plain and a complex mountain system including parts of the Eje Volcánico Transmexicano and the Sierra Madre Oriental [20]; elevations vary from sea level to more than 5000 m [21], [22].

### Study species

Twenty-one species' of venomous snakes are present in Veracruz: 16 vipers and 5 coral snakes [17]. However, we discarded coral snakes for consideration since the potential danger that they present is minimal [23]. We also eliminated rare species' as the probability of encounter of these snakes with humans is low [17], and because presence records for these species' are scarce, which affects performance of ENMs (i.e., [24]–[26]). Therefore, we selected as study species' only vipers with six or more presence records in the state: *Atropoides nummifer*, *Bothrops asper*, *Crotalus atrox*, *C. intermedius*, *C. molossus*, *C. ravus*, *C. simus*, *C. totonacus*, and *C. triseriatus*.

### Ecological Niche Modeling

Occurrence data for the entire geographic range of snake species' were gathered from three sources: 1. Unpublished personal records (these were occasional observations obtained from work activities in wildlife monitoring. No special permits were required because we did not handle the snakes in any way), 2. Specialized literature [17], [27], [28], and 3. Online available information accessible through the Global Biodiversity Information Facility (GBIF; http://www.gbif.org), the World Information Network on Biodiversity (REMIB: www.conabio.gob.mx/remib/doctos/remib_esp.html) and HerpNet (http://www.herpnet.org). Occurrences lacking latitude-longitude coordinates were georeferenced with gazetteers and Google Earth (http://earth.google.es/). Because presence-only data may present sampling bias and spatial autocorrelation that negatively impact model performance [29], we overlaid a 0.05° resolution reticule over the study region and randomly removed duplicates, leaving a single occurrence per grid cell [30]. As well, we removed doubtful and ambiguous occurrences.

Environmental layers regarding climate and topography were used to generate the ENMs (Table S1); climate variables were obtained from the WorldClim database (http://www.worldclim.org) [31] and topographic information was derived from the SRTM elevation model (http://srtm.csi.cgiar.org). All environmental data were standardized to geographic coordinates (Datum WGS-84) at a spatial resolution of 30”. We screened for collinearity by examining pairwise correlations between variables for each species. When a pair had a Pearson product-moment correlation coefficient >0.7, one of the two variables was removed [32]. For each viper, the extent of the layers varied according to the limits of biogeographic provinces [33] containing all its occurrences [34].

Maps of potential distributions for each species were obtained using desktop GARP [35], an evolutionary, computing algorithm that has been tested extensively for predictions of the geographic distributions of species' [36]–[39]. We developed 100 replicate models for each species based on bootstrapped subsamples of available occurrence data. Following Anderson et al. [40] we retained the 10 best models as these having the lowest omission error and lowest departure from the median area predicted suitable. For each species these models were summed in ArcGIS 10 to produce a consensus map [41]. Finally, consensus maps were transformed to produce binary maps using the minimum presence value (MPV) as a threshold criteria, namely the highest raw suitability value at which all input occurrence points were included in the presence area.

In all cases, 80% of presence records were used in model calibration and the remaining 20% were used for model evaluation. We evaluated model performance using a partial ROC approach following Peterson et al. [42], a modification of the area under the curve (AUC), and receiver operating characteristic (ROC) approach [43]. This method avoids some disadvantages of the traditional ROC method [44], and is implemented in a stand-alone software [45].

To characterize ecological niches of vipers and calculate DNC values, we followed Yañez-Arenas et al. [15] and Martínez-Meyer et al. [14]. In brief, we extracted values of environmental variables for all pixels where the species' was predicted present according to the binary potential distribution maps. We standardized each dimension by subtracting the mean and dividing by the standard deviation, producing a standard normal variable (i.e., mean = 0, variance = 1). In this way, the multidimensional niche centroid is the point where values of all variables is 0, and calculations of DNCs become simple decompositions of Euclidean distances. Finally, using the “Zonal Statistics as Table” option in ArcGIS 10 [41], we obtained the mean distance to the niche centroid in each municipality of Veracruz based in a data set of municipalities [46]. Distances were calculated for each municipality because snakebite reports and the socioeconomic variables described in the next section were developed at this level.

### Snakebite incidence and socioeconomic variables

Snakebite reports for each municipality in the period 2003-2012 were obtained through the *Sistema Único Automatizado para la Vigilancia Epidemiológica* (SUAVE; Secretaría de Salud del Estado de Veracruz). In order to reduce variability caused by unequal population size in municipalities, we estimated the smoothed snakebite incidence (expressed per 100,000 inhabitants over this ten year period) using the tool for automated spatial Bayesian smoothing of incidence rates available in SIGEpi v 1.4 [47]. Smoothed incidence was linked to the dataset of municipalities. Separately, we obtained socioeconomic information from INEGI [46] including fields summarizing human population density, an index of marginalization, and percentage of population without health insurance, all of which have previously been considered as associated with snakebites [48], [49].

Generalized additive models (GAMs) with quasi-Poisson family responses were used to evaluate relationships between smoothed snakebite incidence and predictor variables [50]. We first built univariate models and then tested all possible combinations (multivariate models with interactions) of significant variables (P<0.05). All estimated parameter effects for each hypothesis were evaluated by comparing generalized cross-validation (GCV) scores [51]. GAM analyses were performed via the mgcv library [52] in R 2.8.1 [53].

## Results

**Figure 2:**
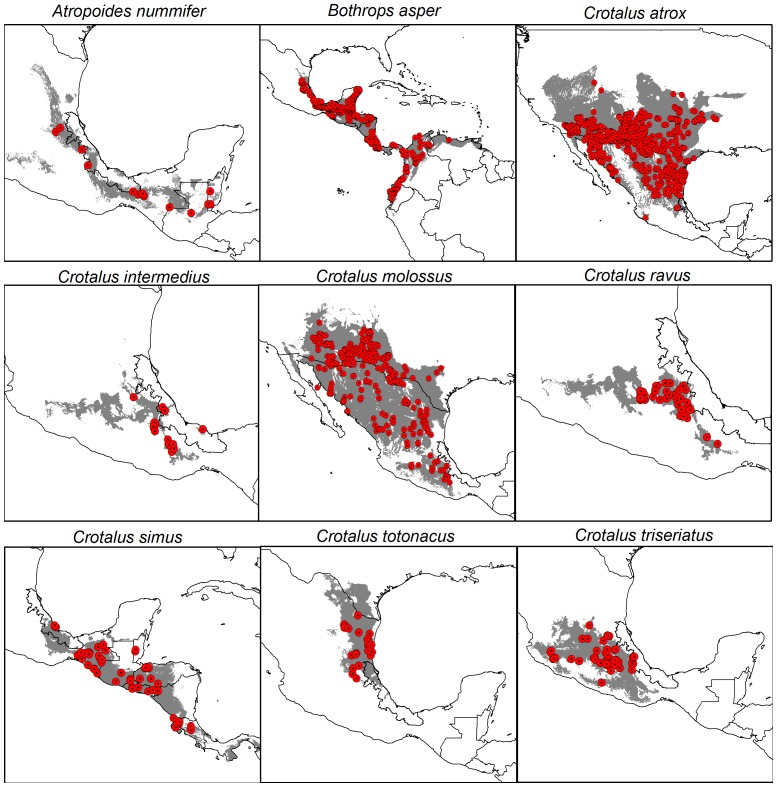
Occurrences and potential distributions of the vipers commonly distributed in Veracruz, Mexico.

We first obtained 2,408 occurrences for viper species' distributed in Veracruz. However, after depuration and filtering there were 1,665 spatially unique localities (Figure 2, Dataset S1). Records were not distributed equally among species': almost 90% belonged to three species (*B. asper*, *C. atrox* and *C. molossus*). Whereas for *A. nummifer* and *C. intermedius*, we could only gather 14 and 15 presences (Table 1).

**Table 1:** Viper occurrence data (N) and Partial ROC analyses results (Mean Ratio = MR, standard deviation = SD, significance = *P*).

		Partial ROC		
Species	N	MR	SD	*P*
*A. nummifer*	14	1.977	0.008	<0.001***
*B. asper*	273	1.406	0.029	<0.001***
*C. atrox*	851	1.262	0.025	<0.001***
*C. intermedius*	15	1.449	0.078	<0.001***
*C. molossus*	336	1.146	0.018	<0.001***
*C. ravus*	41	1.340	0.095	<0.001***
*C. simus*	53	1.660	0.035	<0.001***
*C. totonacus*	24	1.964	0.004	<0.001***
*C. triseratus*	58	1.938	0.029	<0.001***
Total	1665		-	-

*** = <0.001, ** = <0.01, * = <0.05.

**Figure 3:**
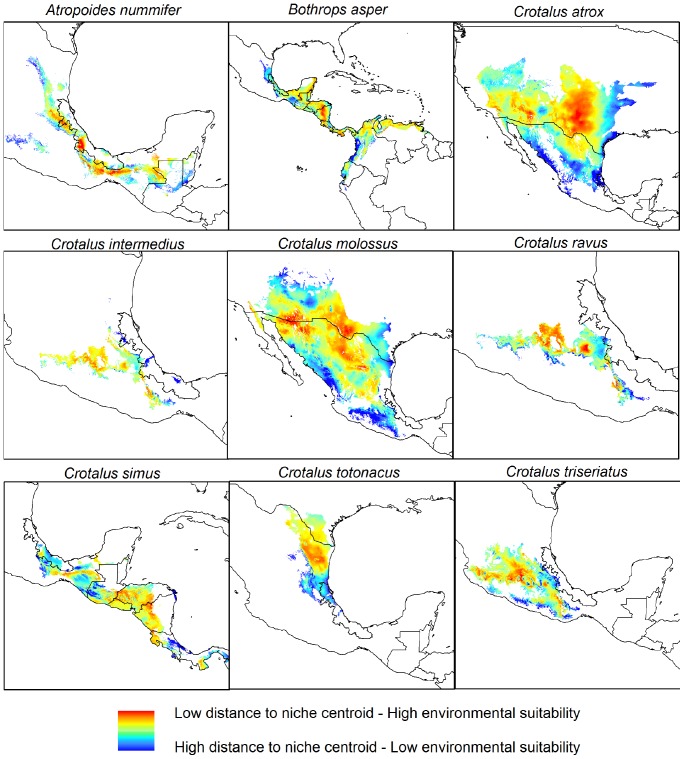
Geographic representation of the distance to niche centroid of the vipers.

Models developed for each species corresponded generally well with knowledge of species' distributions (Figure 2) and their environmental preferences (Figure 3) [16]. Partial ROC analysis exhibited high average AUC ratios and low standard deviations for all models; according to this test, all were significantly better than random (Table 1).

**Figure 4:**
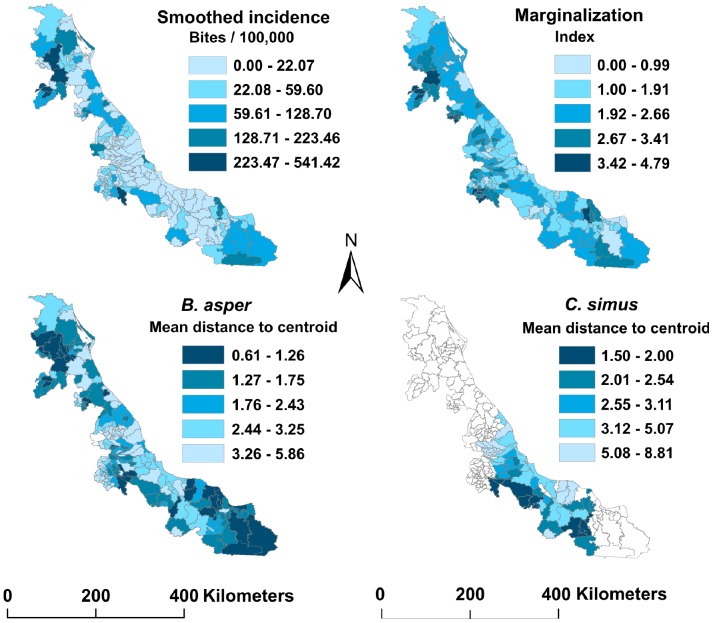
Distribution per municipality of smoothed snakebite incidence (2003–2012), marginalization and distances to niche centroid of *Crotalus simus* and *Bothrops asper*. White municipalities have no data.

A total of 3,765 snakebite cases were reported for Veracruz during 2003–2012, corresponding to an average incidence of 4.93 (±1.30) snakebites per 100,000 inhabitants per year. Municipalities with the highest smoothed incidence corresponded to the northern and southern regions of Veracruz, whereas the central-east region presented the lowest incidence (Figure 4).

**Figure 5:**
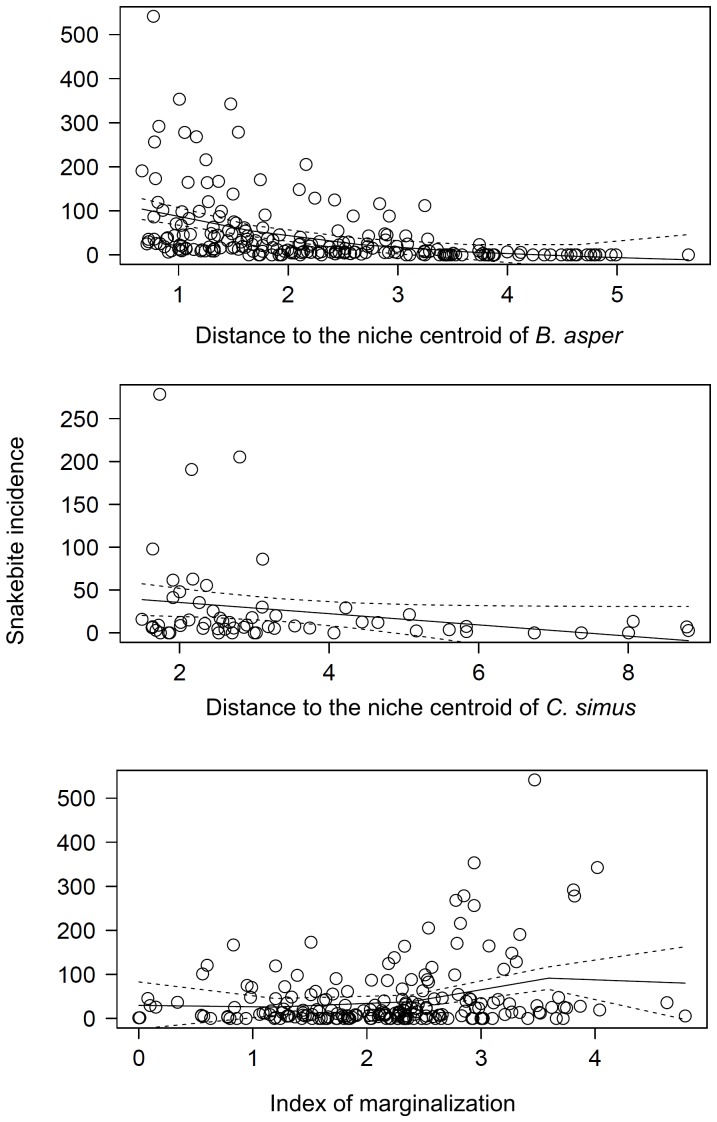
Relationships between snakebite incidence and distances to niche centroid of *Crotalus simus*, *Bothrops asper* and the marginalization index (INEGI 2010).

Univariate generalized additive models showed that DNC of most vipers were negatively correlated with snakebite incidence (all but *C. totonacus* and *A. nummifer*). However, these relationships were significant only for *B. asper* and *C. simus*. The former explained almost 35% of model deviance and the latter 15%. Marginalization of human populations was positive correlated with snakebites, and explained an additional 17% of deviance (Table 2, Figure 5).

**Table 2:** Univariate generalized additive models relating snakebite incidence and explanatory variables.

Variable	*p* value	R^2^ (adj)	DE (%)
DNC of *B. asper*	<0.001***	−0.191	34.9
Index of marginalization	<0.001***	0.101	17.5
DNC of *C. simus*	0.014*	−0.074	15.1
DNC of *C. atrox*	0.063	−0.057	14.3
DNC of *C. intermedius*	0.052	−0.036	13.6
DNC of *C. triseriatus*	0.141	−0.025	11.4
DNC of *C. ravus*	0.606	−0.024	9.3
DNC of *C. molossus*	0.494	−0.017	9.1
Population density (ind/km)	0.302	0.005	3.4
DNC of *C. totonacus*	0.330	0.009	2.7
Population without health insurance (%)	−0.702	−0.008	1.3
DNC of *A. nummifer*	0.677	0.007	0.1

R^2^ (adj) = adjusted coefficient of determination, DE (%) = percentage of deviance explained.

*** = <0.001, ** = <0.01, * = <0.05.

In multivariate models, comparisons of candidate models revealed that DNC for *B. asper* was a very important parameter explaining snakebite incidence, being included in all of the top five best models. The best-fit model explained 76% of deviance and is based on the DNCs of *B. asper* and *C. simus* and marginalization (Table 3).

**Table 3:** Multivariate generalized additive models relating snakebite incidence and explanatory variables.

Model	R^2^(adj)	DE (%)	GCV
1. (csim, marg)+(basp, marg)	0.695	76.0%	24.893
2. csim+basp+marg	0.678	71.7%	25.335
3. csim+basp	0.265	46.1%	37.797
4. basp+marg	0.406	52.6%	41.97
5. (basp, marg)	0.388	53.0%	42.614

R^2^ (adj) = adjusted coefficient of determination, DE (%) = percentage of deviance explained, GCV = generalized cross validation score. Model parameter keys: basp = DNC of *Bothrops asper*, csim = DNC of *Crotalus simus*, marg = marginalization.

## Discussion

Recent developments in the field of ENM and broad availability of rich global environmental data sets have augmented ability to predict distributions of species' related to transmission of diseases [54]–[57]. However, until now, the problem of snakebite has never been addressed. Our results demonstrate that this can be done by mapping potential environmental suitability of vipers through the DNC approach [14], [15].

We found significant inverse relationships between snakebite incidence and DNCs for *B. asper* and *C. simus*. These species' are the main cause of incidents in the region [58], and our results suggest that, when sufficient occurrence information is available, ENM and DNC approaches, offer an alternative approach to understanding snakebite incidence risk. *B. asper* is probably the most dangerous snake in Latin America, because of its broad distribution, size, habits and aggressiveness; also, according to Sasa and Vázquez [18], this viper is well adapted to environments affected by small-scale agriculture, making snake-human encounters frequent during agricultural activities in fields and close to rural dwellings. *C. simus* is not widely distributed in Latin America, but it is quite common in Veracruz and is frequently found in areas of livestock and crops, increasing rates of encounter with humans [17].

For the remaining species', we found no significant relationships between DNC and snakebite incidence. Habitat preferences could be responsible for this lack of relationship, although certainly other factors enter the picture as well (behavior, demography, distribution patterns, among others). *Crotalus molossus*, *C. triseriatus*, *C. ravus* and *C. intermedius* inhabit diverse vegetation types, but principally desert and pine-oak forest [17]; more open vegetation than tropical forests, making it easier to detect the presence of the vipers. *C. totonacus* and *C. atrox* also occur in tropical deciduous forest, but the former is a very uncommon snake and the latter prefers other ecosystems (deserts, mesquite grasslands, scrublands and pine-oak forest) [17]. The probability of encounters between humans and *A. nummifer* is low, because this species inhabits mainly primary well-preserved forests, where anthropogenic activities are scarce [17].

Examination of the shape of the relationships between DNC and snakebite incidence suggest that DNC indicates the upper limit of snakebite incidence rates, rather than the average (Figure 5). An important observation is that several municipalities presented high environmental suitability, but incidences that were nil or very low. That is, according to the abundance-DNC relationship hypothesis, when DNC is low, the species is expected to be abundant; however, diverse factors may affect this relationship, such as microclimate, biotic interactions and dispersal limitations, which may depress abundance in otherwise suitable areas [59]. On the other hand, hospital data may be biased, as has been noted elsewhere in the world [60], [61], especially when incidences are low. Snakebite incidence is frequently underreported owing to lack of effective health infrastructure in marginalized rural communities, and because many cases are not reported because patients either prefer traditional medicine or die before reaching hospitals [62]–[65]. The contrasts between hospital reports and community data suggest that most snakebite victims turn first to traditional healers and only go to hospitals when poisoning is severe and traditional treatments inadequate [48]. Conversely, in some areas adjacent to the big cities the experience and reputation of urban health centers can attract some patients which does not reflect actual local incidence rates. Our use of smoothed incidences to attenuate variability caused by unequal population size in municipalities should help in this regard, however there are many drawbacks in case notification rates that encourage develop of alternative approaches to inferring potential snakebite risk, such as the DNC method; environmental suitability can complement socioeconomic and health-related factors to complete the picture of the phenomenon (see below).

Human population has been considered as inversely correlated with snakebite incidence [48], [66], [67]. This inverse relationship may be explained by both, reduction of snake populations in human populated areas, and changes in human condition and occupation [68], [69]. Activities in rural areas such as agriculture, grazing and fishing significantly increase risk of snake encounter [7], [8]. In Veracruz, we did not observe significant relationships between human population density and snakebite incidence (Table 2). This may be due to the scale of our analysis, the characteristics of human populations, or the presence and abundance of the vipers. For instance, some of the municipalities with lowest human densities in the state are also arid areas, where *B. asper* and *C. simus* are absent or uncommon. Another important social factor is poverty: at global scales, Harrison et al. [49] demonstrated that socioeconomic indicators of poverty correlate with snakebite-induced mortality. We observed a positive correlation between snakebite incidence and marginalization of municipalities in Veracruz, would could reflect a frequent association between the latter and increased manual agricultural activities (Figure 5, Table 2). Regarding health related factors, we did not observe relationships between snakebite incidence rates and percentage of population without health insurance (Table 2). One possible explanation for this result is because municipalities with 100% of inhabitants without health insurance may also lack proper systems of snakebite detection. As such, zero cases in a municipality could be real, or may reflect failures in data collection.

Each of these factors interacts with others, as was demonstrated in this study via the multivariate generalized additive models; consequently, it is important to take all into account for a better understanding of the epidemiological problem that snakebites present. The ENM-DNC method approach, in combination with socioeconomic variables, could help in this task by mapping potential distributions and environmental suitability for dangerous snakes, identifying areas of greater potential risk in which to focus educational and medical remediation in the form of supplies and facilities. These analyses should also be applied elsewhere in the world to evaluate the generality of our findings. A further corroboration of our models would be direct population density estimates for key viper species in areas of differing DNC (although this task is much complicated by the secretive nature of many of the species), and detailed assessment of snakebite incidence through household surveys.

## Supporting Information

Table S1 Variables used in distribution models. Please note that temperature data are in °C * 10, units used for the precipitation data is mm (millimeters), altitude is expressed in masl (meters above sea level) and slope in percentage (%).
(DOCX)

Table S2 Sources for occurrence data of vipers.
(DOCX)

Dataset S1 Databases with the occurrences for all species' modeled. Codes for each database: *Atropoides nummifer* = r_a_num, *Bothrops asper* = r_b_asper, *Crotalus atrox* = r_c_atr, *C. intermedius* = r_c_int, *C. molossus* = r_c_mol, *C. ravus* = r_c_rav, *C. simus* = r_c_sim, *C. totonacus* = r_c_tot, *C. triseriatus* = r_c_tri.
(RAR)

Dataset S2 Cases of snakebites in Veracruz (2003–2012). Raw information obtained through the *Sistema Único Automatizado para la Vigilancia Epidemiológica* (SUAVE; Secretaría de Salud del Estado de Veracruz).
(RAR)
