# Supplementary material for: The Use of Ecological Niche Modeling to Infer Potential Risk Areas of Snakebite in the Mexican State of Veracruz
Source: PLoS One. 2014 Jun 25;9(6):e100957. doi: 10.1371/journal.pone.0100957 (PMC4071012; doi:10.1371/journal.pone.0100957)
Supplement: Table S1 — Variables used in distribution models. Please note that temperature data are in °C * 10, units used for the precipitation data is mm (millimeters), altitude is expressed in masl (meters above sea level) and slope in percentage (%). (DOCX) [file pone.0100957.s001.docx]

Table S1. Variables used in distribution models. Please note that temperature data are in °C * 10, units used for the precipitation data is mm (millimeters), altitude is expressed in masl (meters above sea level) and slope in percentage (%).

| Variable | Source |
| --- | --- |
| BIO1 = Annual Mean Temperature | WorldClim |
| BIO2 = Mean Diurnal Range (Mean of monthly (max temp - min temp)) | WorldClim |
| BIO3 = Isothermality (BIO2/BIO7) (* 100) | WorldClim |
| BIO4 = Temperature Seasonality (standard deviation *100) | WorldClim |
| BIO5 = Max Temperature of Warmest Month | WorldClim |
| BIO6 = Min Temperature of Coldest Month | WorldClim |
| BIO7 = Temperature Annual Range (BIO5-BIO6) | WorldClim |
| BIO8 = Mean Temperature of Wettest Quarter | WorldClim |
| BIO9 = Mean Temperature of Driest Quarter | WorldClim |
| BIO10 = Mean Temperature of Warmest Quarter | WorldClim |
| BIO11 = Mean Temperature of Coldest Quarter | WorldClim |
| BIO12 = Annual Precipitation | WorldClim |
| BIO13 = Precipitation of Wettest Month | WorldClim |
| BIO14 = Precipitation of Driest Month | WorldClim |
| BIO15 = Precipitation Seasonality (Coefficient of Variation) | WorldClim |
| BIO16 = Precipitation of Wettest Quarter | WorldClim |
| BIO17 = Precipitation of Driest Quarter | WorldClim |
| BIO18 = Precipitation of Warmest Quarter | WorldClim |
| BIO19 = Precipitation of Coldest Quarter | WorldClim |
| DEM = Altitude | SRTM |
| SLP = Slope | SRTM |
