# Supplementary material for: The Use of Ecological Niche Modeling to Infer Potential Risk Areas of Snakebite in the Mexican State of Veracruz
Source: PLoS One. 2014 Jun 25;9(6):e100957. doi: 10.1371/journal.pone.0100957 (PMC4071012; doi:10.1371/journal.pone.0100957)
Supplement: Table S2 — Sources for occurrence data of vipers. (DOCX) [file pone.0100957.s002.docx]

Table S2. Sources for occurrence data of vipers.

| 1 | Herpetology Collection - Royal Ontario Museum |
| --- | --- |
| 2 | Field Museum of Natural History (Zoology) Amphibian and Reptile Collection |
| 3 | UTA Herpetology (UTA-A UTA-R) |
| 4 | Biodiversity Research and Teaching Collections - TCWC Vertebrates |
| 5 | "Colección Herpetológica del Museo de Zoología ""Alfonso L. Herrera"", México (MZFC, UNAM)" |
| 6 | NMNH Vertebrate Zoology Herpetology Collections |
| 7 | Vertebrate Palaeontology Comparative Osteology Collection - Royal Ontario Museum |
| 8 | CM Herps Collection |
| 9 | KUBI Herpetology Collection |
| 10 | UMZC Zoological Specimens |
| 11 | "Museum of Comparative Zoology, Harvard University" |
| 12 | SysTax - Zoological Collections |
| 13 | CUMNH Herpetology Collection |
| 14 | SMNS Herpetologie |
| 15 | MVZ Herp Collection (Arctos) |
| 16 | "Coleccion de Herpetologia, MZFC-UNAM" |
| 17 | LACM Vertebrate Collection |
| 18 | MPM Herpetology (H) |
| 19 | RBINS collections |
| 20 | CAS Herpetology (HERP) |
| 21 | Colección científica del Museo de Historia Natural Alfredo Dugés |
| 22 | Collection Herpetology SMF |
| 23 | Collection Herpetologie - SNSD |
| 24 | CUMV Reptile Collection |
| 25 | Peabody Herpetology DiGIR Service |
| 26 | "Vertebrados terrestres del Corredor Biológico Sierra Madre del Sur, Chiapas, México" |
| 27 | Actualización y enriquecimiento de las bases de datos del proyecto de evaluación y análisis geográfico de la diversidad faunística de Chiapas |
| 28 | "Sistematización de las colecciones científicas del Instituto de Historia Natural y Ecología, (IHNE) Chiapas" |
| 29 | Rapid Assessment Program (RAP) Biodiversity Survey Database |
| 30 | "Computarización de las colecciones de vertebrados terrestres de la Escuela Nacional de Ciencias Biológicas, IPN - Fases 3" |
| 31 | "Anfibios y reptiles del municipio de Cuetzalan del Progreso, Puebla" |
| 32 | Computarización de las Colecciones de Anfibios y Reptiles del Centro de Investigaciones Biológicas de la Universidad Autónoma del Estado de Hidalgo |
| 33 | New Mexico Biodiversity Collections Consortium database |
| 34 | Animal Sound Archive |
| 35 | Herp Collection |
| 36 | UTEP Vertebrates |
| 37 | Georgia Southern University - Savannah Science Museum Herpetology Collection |
| 38 | UCMP Vertebrate Collection |
| 39 | "Zoological Museum Amsterdam, University of Amsterdam (NL) – Herpetology" |
| 40 | Ditsong Museum |
| 41 | Anfibios y reptiles del estado de Nuevo León |
| 42 | UWBM Herpetology Collection (Arctos) |
| 43 | NRM-Herpetology |
| 44 | Inventario nacional de especies vegetales y animales de uso artesanal |
| 45 | Colección de anfibios y reptiles del Laboratorio de Herpetología de la Universidad del Valle (UV-C) |
| 46 | Lund Museum of Zoology (MZLU) |
| 47 | Amphibians and Reptiles collection at the Natural History Museum of Denmark (SNM) |
| 48 | UNSM Vertebrate Specimens |
| 49 | Cowan Tetrapod Collection - Herpetology |
| 50 | SBMNH Vertebrate Zoology |
| 51 | Zoology (Museum of Evolution - Uppsala) |
| 52 | PSM Vertebrates Collection |
| 53 | Bishop Museum Natural Sciences Data |
| 54 | Museu de Ciències Naturals de Barcelona: MCNB-Cord |
| 55 | Macaulay Library Audio and Video Collection |
| 56 | Western Australian Museum provider for OZCAM |
| 57 | "Anfibios y Reptiles del Estado de Tamaulipas, México (UANL)" |
| 58 | "Herpetile collection, Natural History Museum, University of Oslo" |
| 59 | "Computarización de las colecciones de vertebrados terrestres de la Escuela Nacional de Ciencias Biológicas, IPN Fase 1: Estado de México, Hidalgo, San Luis Potosí y Tlaxcala" |
| 60 | "Computarización de las colecciones de vertebrados terrestres de la Escuela Nacional de Ciencias Biológicas, IPN - Fases 2" |
| 61 | iNaturalist research-grade observations |
| 62 | Paleobiology Database |
| 63 | Borror Laboratory of Bioacoustics |
| 64 | UNR Herpetology |
| 65 | "Respaldo de las colecciones de tejidos del Museo de Zoología, Departamento de Biología Evolutiva, Facultad de Ciencias, UNAM (Herpetofauna)" |
| 66 | "Historia natural del parque ecológico estatal de Omiltemi, Chilpancingo, Guerrero, México" |
| 67 | Inventario herpetofaunístico del valle semiárido de Tehuacán-Cuicatlán (continuación) |
| 68 | Inventario herpetofaunístico del valle semiárido de Tehuacán-Cuicatlán |
| 69 | Herpetofauna de la reserva de la biósfera Valle de Tehuacán-Cuicatlán (etapa final) |
| 70 | Reptilia Collection of Museum and Institute of Zoology PAS |
| 71 | "Fonoteca Zoológica, Museo Nacional de Ciencias Naturales, Madrid: FZ_REPTILIA" |
| 72 | Análisis de la heterogeneidad ambiental y conectividad de las áreas naturales del sur del Valle de México_2 |
| 73 | Herpetofauna del corredor biológico Chichinautzin y la Sierra de Huautla en el estado de Morelos |
| 74 | Propuesta para la realización de 37 fichas biológicas de las especies de herpetofauna incluidas en la NOM-059 presentes en la Península de Yucatán |
| 75 | Colección Nacional de Herpetología - Museo Argentino de Ciencias Naturales 'Bernardino Rivadavia' |
| 76 | Fundación Miguel Lillo - Colección Herpetológica |
| 77 | "Museo Nacional de Ciencias Naturales, Madrid: MNCN_Herpeto" |
| 78 | Coleccion Herpetologica Reptiles LJAMM-CNP |
| 79 | University of Ghent - Zoology Museum - Vertebratacollectie |
| 80 | Reptilia ZMK |
| 81 | Colección de Ofidios Museo de La Salle Bogotá (MLS) |
| 82 | Colección de Reptiles del Instituto Alexander von Humboldt |
| 83 | "Vertebrados terrestres del parque nacional Cañón del Sumidero, Chiapas, México" |
| 84 | Formación de la colección de referencia de anfibios y reptiles de la Reserva de la Biosfera de Sian Ka'an |
| 85 | "Anfibios, reptiles y mamíferos del corredor biológico del norte de Yucatán depositados en las colecciones de la Escuela Nacional de Ciencias Biológicas" |
| 86 | "Inventario florístico y faunístico del Parque Nacional Barranca del Cupatitzio, Michoacán" |
| 87 | Vertebrados silvestres en zonas indígenas de la reserva de la biósfera Mariposa Monarca: anfibios y reptiles |
